# The stigma associated with bereavement by suicide and other sudden deaths: A qualitative interview study

**DOI:** 10.1016/j.socscimed.2017.12.035

**Published:** 2018-02

**Authors:** Alexandra L. Pitman, Fiona Stevenson, David P.J. Osborn, Michael B. King

**Affiliations:** a UCL Division of Psychiatry, 6th floor, Maple House, 149 Tottenham Court Road, London, W1T 7NF, United Kingdom; b Camden and Islington NHS Foundation Trust, St Pancras Hospital, 4 Saint Pancras Way, London NW1 0PE, United Kingdom; c UCL Research Department of Primary Care & Population Health, UCL Medical School, Royal Free Campus, Rowland Hill St, London NW3 2PF, United Kingdom

**Keywords:** Stigma, Suicide, Bereavement, Grief, Social support, Taboo, United Kingdom

## Abstract

Quantitative studies have found that suicide bereavement is associated with suicide attempt, and is perceived as the most stigmatising of sudden losses. Their findings also suggest that perceived stigma may explain the excess suicidality. There is a need to understand the nature of this stigma and address suicide risk in this group. We aimed to describe and compare the nature of the experiences of stigma reported by people bereaved by suicide, sudden unnatural death, and sudden natural death, and identify any commonalities and unique experiences. We conducted a population-based cross-sectional survey of 659,572 staff and students at 37 British higher educational institutions in 2010, inviting those aged 18–40 who had experienced sudden bereavement of a close contact since the age of 10 to take part in an on-line survey and to volunteer for an interview to discuss their experiences. We used maximum variation sampling from 1398 volunteer interviewees to capture a range of experiences, and conducted individual face-to-face semi-structured interviews to explore perceptions of stigma and support. We continued sampling until no new themes were forthcoming, reaching saturation at n = 27 interviews (11 participants bereaved by suicide). We employed thematic analysis to identify any distinct dimensions of reported stigma, and any commonalities across the three groups. We identified two key themes: specific negative attitudes of others, and social awkwardness. Both themes were common to interviewees bereaved by suicide, sudden unnatural death, and sudden natural death. All interviewees reported the experience of stigmatising social awkwardness, but this may have been experienced more acutely by those bereaved by suicide due to self-stigma. This study provides evidence of a persistent death taboo in relation to sudden deaths. There is potential for anti-stigma interventions to reduce the isolation and social awkwardness perceived by people bereaved suddenly, particularly after suicide loss.

## Highlights

• Explores stigma after suicide, unnatural deaths and sudden natural deaths.
• First qualitative study internally comparing these three groups' experiences.
• All three groups described stigmatising social awkwardness.
• The death taboo in relation to sudden deaths is particularly marked after suicide.

## 1 Introduction

Although sociologists argue that the death taboo has been exaggerated, and that discussing death is now a relatively normal part of contemporary social discourse (Walter, 1991), societal reactions to suicide suggest that this taboo persists. Stigmatisation of those who die by suicide and their relatives is linked to historical religious, legal and social sanctions against suicide, including its relatively recent decriminalisation (Cvinar, 2005). Whilst any sudden death might be perceived as shocking by its unexpected nature, suicide has long been thought to be the most stigmatising of bereavements. In contemporary society this stigma is thought to arise primarily from social distaste and disapproval, associations of blame and shame, and also from social unease (Chapple et al., 2015, Cvinar, 2005), although few studies have investigated this. Our empirical work on perceived stigma (the subjective awareness of others' stigmatising attitudes) has found suicide bereavement to be the most stigmatising of sudden losses (Pitman et al., 2016b), and suggests that higher stigma scores may partially explain the associations between suicide bereavement and negative outcomes such as suicide attempt (Pitman et al., 2016a), poorer occupational functioning (Pitman et al., 2016a), reduced informal support (Pitman et al., 2017a), and delays in accessing support (Pitman et al., 2017b). Our work has also found an association between the stigma of sudden bereavement and suicide attempt (Pitman et al., 2017a). Given the influence of stigma on help-seeking for mental disorders (Schomerus and Angermeyer, 2008), such findings identify stigma and help-seeking as potential mediators of suicide risk after suicide bereavement. Understanding these mechanisms is a public health priority. Suicide prevention strategies in many high-income countries recommend providision of support for people bereaved by suicide, in view of their suicide risk (Pitman et al., 2014), but lack an evidence base for intervening (McDaid et al., 2008). A better understanding of the role of stigma in creating barriers to uptake of support in this group (Pitman et al., 2017a, Pitman et al., 2017b, Pitman et al., 2016a, Pitman et al., 2016b) would inform service changes to benefit them.

Quantitative differences in stigma scores provide one way of understanding how bereaved people perceive discriminatory attitudes after a loss and how this varies by cause of death. However, they convey little of the nature of those experiences. It is possible that the nature of stigmatising experiences is similar for all those bereaved suddenly, but experienced most acutely after suicide. Alternatively, it is possible that stigmatising attitudes towards suicide loss are unique and more upsetting in their nature. This question of nature *versus* degree requires further investigation using qualitative methods. Such work would help understand how stigma affects help-seeking intentions and behaviour after different kinds of loss. Previous qualitative studies of the experience of suicide bereavement have identified strong perceptions of stigma, the characteristics of which differ according to cultural settings (Cvinar, 2005, Hanschmidt et al., 2016). However, no previous studies have compared experiences of stigma after suicide bereavement to experiences of stigma after sudden natural deaths. Such work could identify how aspects of stigma specific to suicide bereavement might influence suicidal behaviour. Our objective was to conduct a three-way comparison of the experiences of stigma reported by people bereaved by suicide, other sudden unnatural death, and sudden natural death, to identify any commonalities and unique experiences in the dimensions of stigma described. We wished to explore whether it is the violence or unnatural nature of a death that so discomforts others, whether this is specific to suicide, or whether discomfort is generalised to all sudden deaths. In this study we chose to focus on young adults as an under-researched group, given policy concerns about their vulnerabilities to suicide (Pitman et al. 2012), the potential role of stigma in explaining non-help-seeking (Biddle et al., 2007), and their priority status within United Kingdom suicide prevention strategies.

## 2 Method

### 2.1 Methodological approach

Our research questions were: What is the nature of the stigma perceived by people bereaved by sudden causes of death? Does the nature of stigmatising experiences differ by cause of sudden death? We therefore chose to focus on accounts of perceived (felt or subjective) stigma, as distinguished from the public or personal stigma enacted in societal or individuals' avoidance or discrimination (Gray, 2002, Rusch et al., 2014). We acknowledged that interviewees' perceptions of stigma might also be a reflection of self-stigma, and therefore mutually reinforcing (Gray, 2002, Rusch et al., 2014). We chose to use the perspective of critical realism, which distinguishes three domains (empirical, actual, and real) within the reality of the bereaved (Scambler and Higgs, 2001). Our focus was on what this perspective terms the ‘empirical’: the way a social interaction is experienced and interpreted by the bereaved. This avoided the problems of observer bias in trying to capture the ‘actual’; an objective account of how the interaction occurred, gained by observing encounters between bereaved and non-bereaved. It also avoided the issues of recall bias (Range and Thompson, 1987, Wagner and Calhoun, 1991) and social desirability bias (Thompson and Range, 1992) in measuring the ‘real’; the underlying attitudes or intent of the non-bereaved people involved in that interaction.

### 2.2 Study design and participants

We followed COREQ guidelines on the design and reporting of qualitative research (Tong et al., 2007). We employed a mixed methods survey design to collect and analyse qualitative interview data from a nested sample of bereaved adults, drawn from a wider sample of bereaved adults providing quantitative and qualitative data in an online survey. We used a cross-sectional survey design to invite all young adults working or studying at the 164 United Kingdom (UK) higher education institutions (HEIs) in 2010 to participate in a closed online survey to investigate “the impact of sudden bereavement on young adults”. We considered this sampling frame to provide the most efficient, comprehensive and pragmatic means of recruiting a hard-to-reach population of young adults (Pitman et al., 2015), while simultaneously minimising traditional biases associated with recruiting a help-seeking sample.

Our sampling strategy has previously been described in our quantitative work (Pitman et al., 2016a, Pitman et al., 2016b, Pitman et al., 2017a, Pitman et al., 2017b). Briefly, 37/164 (23%) HEIs agreed to take part, providing an estimated sampling frame of 659,572 staff and students. Inclusion criteria were: people aged 18–40 (to define a young adult age range) who had experienced sudden bereavement of a close friend or relative since the age of ten. Early childhood bereavements were excluded to minimise recall bias. Sudden bereavement was operationalised as “a death that could not have been predicted at that time and which occurred suddenly or within a matter of days”. Exposure status was sub-classified, via self-report, as: bereavement by suicide, bereavement by sudden natural causes (eg. cardiac arrest), and bereavement by sudden unnatural causes (eg. accidental death).

The survey elicited on-line responses to a series of closed and open questions. The quantitative (Pitman et al., 2016a, Pitman et al., 2016b, Pitman et al., 2017a, Pitman et al., 2017b) and qualitative data (Pitman et al., 2017c) collected in this questionnaire have been analysed separately. A final question invited respondents to volunteer for a face-to-face interview “to hear more about your personal experiences of bereavement”. From respondents who volunteered online for an interview, we selected a purposive maximum variation sub-sample to reflect a broad range of experiences. This represented a balance of gender, age, ethnicity, geographical location, age at bereavement, time elapsed since bereavement, kinship to the deceased, and cause of death.

### 2.3 Procedures

We developed a topic guide for the semi-structured interviews (Appendix 1), to cover a range of domains impacted after bereavement. This was based on the published research and policy literature (Cvinar, 2005, Public Health England & National Suicide Prevention Alliance, 2015, Sveen and Walby, 2008), and the suggestions of an advisory group of young bereaved adults and bereavement counsellors. Information sheets sent to potential interviewees explained that the purpose of the study was to explore further the impact of the bereavement on everyday life, including how other people had reacted to them because of the loss. Using prompts from each interviewee's online responses, views and specific examples were elicited on topics such as: the attitudes and responses of friends, colleagues and relatives; whether information about the death had been concealed; whether the deceased was still discussed; and how readily support had been offered. We also elicited views on helpful and unhelpful experiences of support, the results of which are being analysed separately.

Participants were interviewed sequentially in university offices in four geographical centres (Belfast, Cardiff, Edinburgh, London) until saturation of themes was reached. Interviews were conducted by the lead author, who was a psychiatrist trained to manage any distress observed. Her only previous contact with interviewees constituted emails determining location and timing of interview. All interviewees gave informed consent at the start of the interview, and were provided with a list of bereavement support organisations. Interviews lasted between 30 and 77 min and were digitally recorded. The topic guide was revised iteratively between interviews, but no repeat interviews were conducted. Field notes were used only to assist transcribing. Instructions were clear that interviewees could terminate or pause the interview at any point. All travel costs were reimbursed but no other participant payment was made. Given the sensitive nature of the topic, transcripts and coded data were not returned to participants for comment unless requested. None was requested.

The study was approved by the UCL Research Ethics Committee in 2010 (ref: 1975/002).

### 2.4 Analytic approach

Interview transcripts were transcribed either by the interviewer (AP) or an independent medical research transcriber. All were checked against original audio by AP to enhance familiarisation with the data. A thematic analytic approach was chosen (Braun and Clarke, 2006) to explore the nature of any experiences or perceptions of stigma in people bereaved by suicide, sudden unnatural death, and sudden natural death. This involved an inductive approach using QSR NVivo 10 to derive analytic categories from reported experiences and perceptions. Two researchers (AP & an independent research consultant) conducted independent thematic analyses of the 27 transcripts. We discussed coding and interpretation of results to explore differences in interpretation of narratives, improve consistency of coding, and reduce the influence of personal reflexivity. The lead author then combined the coded data, to provide rigor in terms of refining the hierarchy of themes and understanding data. A funnelling approach (Burnard, 1991) was used to collapse codes into key themes in discussion with a senior qualitative researcher (FS), and distil codes into higher-order categories. Themes were checked back against the 27 transcriptions to ensure consistency and validity.

## 3 Results

### 3.1 Response

A total of 5085 of the 659,572 people sampled responded to the questionnaire by clicking on the survey link, with 4630 (91%) consenting to participate in the online study, and 1398 (30%) volunteering for a further face-to-face interview.

**Fig. 1:**
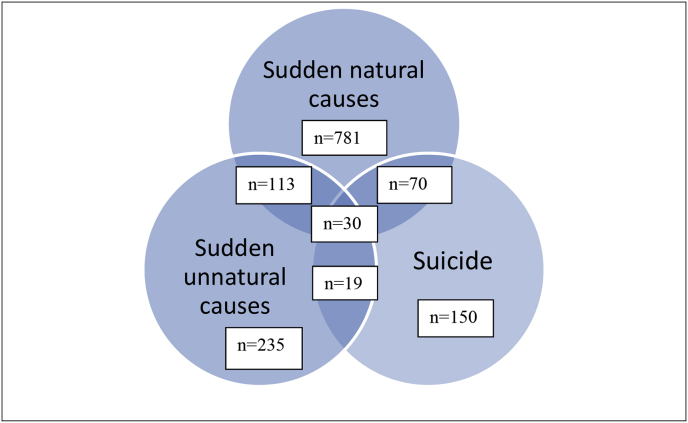
Bereavement exposure in all participants volunteering for interview (n = 1398).

The majority of the interview volunteers had been bereaved by sudden natural causes (Fig. 1), and the smallest category comprised those bereaved by suicide. Overall, 232 volunteers had experienced more than one mode of sudden bereavement, and this was more common an experience in the group bereaved by suicide.

### 3.2 Participant characteristics

Saturation of themes was achieved once 27 respondents had been interviewed (nine men and 18 women). In this sample 11 interviewees reported having been bereaved by the suicide of a close contact (of whom one had also been bereaved by sudden natural causes), 6 had been bereaved by sudden unnatural causes (of whom one had also been bereaved by sudden natural causes), and 10 had been bereaved by sudden natural causes.

The 27 participants ranged in age from 20 to 40. The majority indicated white ethnicity (93%), single status (52%), co-habitation with relatives or friends (89%), and a bereavement that had occurred two or more years previously (78%). Most (78%) were students in higher education, whilst the remainder were HEI staff. All were UK residents, with 20 (74%) of British nationality, 2 from Eastern Europe; 2 from Southern Europe; 2 from the Republic of Ireland, and 1 from North America. Of British interviewees, 6 were from Northern Ireland (denoted by * in quotes below), a region with a history of violent conflict and high suicide rates (Office for National Statistics, 2017).

### 3.3 Themes identified

We identified two main themes in relation to the stigma described by interviewees: specific negative attitudes, and social awkwardness (Table 1).

**Table 1:** Themes of stigma identified in analysis of n = 27 interview transcripts.

Higher-order theme	Mode of bereavement	Bereaved by suicide	Bereaved by sudden unnatural death	Bereaved by sudden natural death
	Sub-theme			
Specific negative attitudes of others				
	Blame	√	√	
	Morbid fascination	√	√	√
	Pity	√	√	√
Social awkwardness				
	Disrupted interactions	√	√	√
	Aversion to displays of grief	√	√	√
	Avoidance of the topic	√	√	√
	Avoidance of the bereaved	√	√	√
	Failure to offer support	√		
	Avoidance of the word suicide	√		
	Concealment of the cause	√	√	
	Tension over disclosure	√	√	√

#### 3.3.1 Specific negative attitudes of others

Just under half of interviewees described experiences of others’ negative attitudes, which separated out into three sub-types.

##### 3.3.1.1 Blame

Examples of people experiencing judgemental attitudes, either towards the deceased or the bereaved, were rare and only arose from interviewees bereaved by suicide or by other sudden unnatural death. The latter group described the deceased being blamed for their risky behaviour.

“And I think that a lot of people thought that he probably died because he could be careless, and it was you know, the things like driving like a nutter or..... I don't think it was a great shock to some of his friends that he died, doing something risky or something sort of high speed, you know, ice-climbing … I just kind of felt like everybody just gets tarred with the same brush.” *(B2 – 37 year old British woman bereaved 5 years previously by sudden unnatural death of uncle)*

Some people bereaved by suicide, predominantly non-British interviewees, perceived that others blamed them and other relatives or friends as responsible for having driven the deceased to suicide:

“the accusation … is that (*my aunt*) actually pushed (*my uncle*) to suicide… it was a discussion that was happening a lot behind doors, …I remember discussions between … my dad and my mum saying how much she handled (*his financial problems*) wrong”. *(A1 - 32 year old Southern European woman bereaved aged 15 by suicide of uncle)*

“(*my uncle, aunt and grandmother*) were constantly repeating that my mother had killed him” *(C5 – 35 year old Eastern European woman bereaved aged 15 by suicide of father)*

“There were people gossiping saying that …. we had videotaped him doing it, like some sort of Satanic ritual or something”. (C4 - 22 year old British* man bereaved aged 16 by suicide of friend)

##### 3.3.1.2 Morbid fascination

Interviewees in all three groups reported being distressed by others’ morbid fascination with the death. This was primarily described in relation to acquaintances rather than close friends. Their insensitive questions appeared to be borne out of morbid curiosity and a desire to report gossip, rather than reflecting genuine concern.

“I felt people were treating it like gossip … and I just thought, how insensitive can you be? … just because the person is not directly related to them, then people think they can just talk about it and tell other people” *(B1 - 20 year old British woman bereaved 6 months previously by suicide of friend)*

“Everybody just wanted to know what happened and … you can tell the difference between people who genuinely knew him …. or people that just wanted to be nosey …. just to give them something to talk about! (C12 - 20 year old British* woman bereaved 7 months previously by sudden natural death of grandfather)

##### 3.3.1.3 Pity

A strong aversion to being pitied was described by interviewees in all three groups, but most markedly in those bereaved by natural causes. They perceived pity in expressions of false sympathy, and felt looked down upon or set apart:

“because it wasn't really something that happened all the time … It sort of made you stand out a bit …. and again, you know, the feeling that people were pitying you or talking about you … I think things like this was where the stigma came from”. (C11 – 37 year old British* woman bereaved aged 13 by sudden natural death of father)

#### 3.3.2 Social awkwardness

All 27 interviewees reported that their bereavement had caused widespread social embarrassment and discomfort, characterised by their own and others’ uncertainty over the social rules influencing interactions after a sudden loss. This reinforced their own sense of social awkwardness and placed a strain on relationships.

##### 3.3.2.1 Disrupted interactions

Other people's awareness of a person's history of sudden bereavement appeared to create a fear that mentioning the topic might open “a real can of worms”, and this disrupted social interactions for interviewees in all three groups. The task of negotiating such awkward social interactions reinforced a sense of difference:

“I just really don't like the whole stepping on eggshells around me or being careful; I'd just rather they act as normal”. (C7 - 25 year old British* woman bereaved aged 21 by suicide of father)

“I think people would express sympathy and say, you know, “Are you OK?” because they felt that that was the natural thing to do, …but … there is that worry that it’s going to be a real can of worms, and what do you do if … they start talking about the death and they cry.… They therefore don't really want to go there, because they're quite frightened and also they don't want the burden of it … I suppose you worry that … there are certain subjects that they can't bring up. So, everybody has a moan about their parents … and you see people hesitating, because they kind of think, “Oh! Can I talk about my own life, because I know that you don't have that, and is that going to upset you?” *(A2 - 29 year old British woman bereaved aged 22 by sudden natural death of father)*

##### 3.3.2.2 Aversion to displays of grief

Many had learned to hide their grief because they felt that other people found outpourings of grief deeply embarrassing. They described social expectations to recover quickly, with any signs of prolonged grief regarded by others as over-reaction to an event they should have ‘got over’ within months.

“You just learn to shut it down, put a smile on.” *(B2 – 37 year old British woman bereaved 5 years previously by sudden unnatural death of uncle)*

“(*hiding my grief is*) more to do with that embarrassment thing, it's like that there's something wrong with me for still feeling upset about it. I think it's that I think they'll think that I'm over-reacting.” *(C6 – 32 year old Irish woman bereaved 11 years previously by sudden accidental death of a friend)*

“I avoid people because if anyone asks me how I am, I don't want to lie and be like, “Oh, I'm fine!”, which is what everyone does.” (C12 - 20 year old British* woman bereaved 7 months previously by sudden natural death of grandfather)

##### 3.3.2.3 Avoidance of the topic

Interviewees from all three groups described a widespread avoidance of the topic of the bereavement. This was interpreted as others' discomfort over the nature of the death, not knowing what to say, their efforts to avoid awkwardness, and fear of emotional outbursts or someone ‘getting heavy’.

“for a lot of people, the idea of talking about death in public at all, it's just not … you're not supposed to talk about sad things, because socialising is about being happy.” *(A4 – 32 year old British woman bereaved 1 year previously by suicide of friend)*

“most of them just sort of go a little bit quiet and don't really want to talk about it” *(D5 – 30 year old British man bereaved 7 months previously by sudden accidental death of friend)*

“People don't know what to say and I understand that, but at the same time, it is awkward, and it feels sort of, you know, rude nearly when people don't say …. “I was sorry for your loss.” or “How are you?” even, or anything.” *(C1 – 31 year old Irish woman bereaved 18 months previously by sudden natural death of mother)*

“I think … people don't know how to deal with emotion at the end of the day. It's human nature … I don't think it's malicious. I don't think they're consciously trying to be hurtful, or be unhelpful or not be helpful; I think it's just fear, I really do. … it's avoiding a scene, it's avoiding, you know, the display in public of emotions; very British” *(D5 – 30 year old British man bereaved 7 months previously by sudden accidental death of friend)*

These experiences seemed more extreme for people bereaved by suicide, because of others’ specific discomfort and distaste over the notion of suicidal deaths:

“with suicide, people, even after the initial shock, didn't want to talk about it. They don't like to acknowledge these things happening …. basically I think it's that fear of not knowing how to approach the topic and not approaching the topic. It's quite bizarre”. *(D7 - 27 year old British man bereaved aged 17 by suicide of mother)*

The consequence of avoiding the topic, particularly after suicide, left the bereaved feeling neglected.

“it was never brought up, ever *(slight laugh)*. Which I didn't really appreciate, because I didn't want to bring it up … I would have preferred if *(my housemates)* had asked, because ….I felt like they just forgot, or didn't really care” *(A5 - 20 year old British woman bereaved 2 years previously by suicide of friend)*

The bereaved frequently avoided the topic themselves, either to prevent themselves and others from feeling awkward, or as a form of self-protection; helping them contain their emotions, or conceal the cause of death.

“I didn't want to bring it up because whenever I said anything, people kind of, they looked a bit awkward about it and … it just makes it a really awkward situation from then on; erm, like … you can't have a proper conversation *(D6 - 22 year old British man bereaved aged 17 by sudden natural death of mother)*

When both parties avoided the topic, opportunities were missed to provide support:

“I broached the subject years, maybe about five years ago with my friend and said, …that I thought I couldn't … that if I brought it up it was like a taboo issue …. with this very good friend, and she was astonished that I felt like that and she said, no, it was that she just didn't always know how to talk about it. “ *(C6 – 32 year old Irish woman bereaved aged 21 by sudden accidental death of a friend)*

Such examples suggested that self-stigma played a key role in compounding the social awkwardness experienced by others in the context of any difficult subject.

##### 3.3.2.4 Avoidance of the bereaved

Interviewees often felt that others avoided them due to the awkwardness of the topic and the interaction in general.

“people … just didn't know how to deal with it, so they just sort of stayed away”. (*C2 - 25 year old North American woman bereaved aged 16 by sudden natural death of father*)

“my other close friend, she avoided me, she did, at first. She didn't know what to say, she didn't know what to do and I don't think she understood that I couldn't just shake myself out of it”. *(D2 - 36 year old British woman bereaved 2 years previously by sudden natural death of mother)*

“I met one of my Mum's friends in (*a supermarket*) car park not long after (*my uncle*) had died and she said she'd only just heard and she was desperately sorry, and …. my eyes teared up and I got a bit upset and she just sort of patted my arm and said ‘Oh, ….I don't mean to stop you’ and … she went off to her car, and I was left sort of standing in the car park” *(B2 – 37 year old British woman bereaved 5 years previously by sudden unnatural death of uncle)*

##### 3.3.2.5 Failure to offer support

Avoidance of the bereaved was experienced *in extremis* by four interviewees, all bereaved by suicide, who interpreted a complete lack of offers of support from friends, family and health professionals as indicating that “no-one wanted to know”. This avoidance of offering support was sometimes understood as being driven by not knowing what to say, but was experienced as stigmatising. Individuals felt slighted, sometimes to the point of outrage, by how unresponsive people could be after such a traumatic event.

“I don't think very many people said anything (*on returning to work after father's suicide*), except, “It's nice to see you back.” A few people said that, and I was working in a building of about 120 staff, six of which were women, and it was women that came out and said, “It is nice to see you back,” and none of the men …. I suppose I thought, like, “I've lost my dad, and you can't say “Hello”?” You know, it was like I was angry that they'd forgotten about it.” (C7 - 25 year old British* woman bereaved aged 21 by suicide of father)

“Well, there was no information about counselling, no bereavement counselling, which I thought, you know, looking back, would be the first thing that someone would be doing …. I don't even think that you even need to be a doctor *(slight laugh)* to give that sort of advice and, there was just nothing, absolutely nothing! And I felt … the stigma of the suicide, really, just that no-one wanted to know.” *(C9 - 40 year old British man bereaved aged 16 by suicide of brother)*

The psychological value of being offered support was stressed repeatedly, with the sense that this communicated social acceptance.

“personally I would really appreciate … just knowing that someone has offered (*support*) … and that there is the opportunity to talk to someone, if I want to”. *(A1 - 32 year old Southern European woman bereaved aged 15 by suicide of uncle)*

##### 3.3.2.6 Avoidance of the word suicide

People bereaved by suicide described the use of the word suicide as having a particularly disruptive effect on conversations. This was described by one interviewee (*bereaved by non-suicide death*) as “a fairly violent word”, and those bereaved by suicide had learned to avoid using it.

“I think there's always the shock factor. It doesn't matter how long afterwards it is, people always … there is quite a lot of shock. You say it and there is that silence. People really don't know what to say …. I think normally, in my experience, I end up having to fill that silence. It normally needs me to change the topic area.” *(D7 - 27 year old British man bereaved aged 17 by suicide of mother)*

“I think people really, really don't like you saying suicide …. It makes other people uncomfortable I think …. I think it's a lot easier to have a conversation with someone about the death of their grandma, who's … passed away in her sleep, or whatever, I think, because it's just less of an awkward topic … and you don't have to try and think about someone's intentions”. *(A5 - 20 year old British woman bereaved 2 years previously by suicide of friend)*

##### 3.3.2.7 Concealment of the cause

People bereaved by suicide or other unnatural deaths described the strain of maintaining secrecy over the true cause of the death. They avoided discussing the death for fear that the truth would threaten a relationship. These interviewees were predominantly from non-British European countries, and their reasons for concealment related to an anticipation of blame, horror, or morbid curiosity. This theme therefore linked to the anticipated negative attitudes of others.

“I remember, I was really, really surprised because I've always thought that the only people who knew was my closest family … and then, after a week, I remember I found out that my uncle and my auntie know, and my cousins, and I was so shocked …” *(C8 – 29 year old Eastern European woman bereaved 2 years previously by sudden unnatural death of partner)*

“even my husband doesn't know how (*my father*) died … I said that he died through a car accident.”. *(C5 – 35 year old Eastern European woman bereaved aged 15 by suicide of father)*

##### 3.3.2.8 Tension over disclosure

Even years after the event, disclosing the unnatural death of a relative or partner to a new partner or friend was associated with significant anxiety due to a fear of rejection. Past experience of rejection after dropping the ‘bombshell’ had reinforced this anxiety.

“that friendship just ended there, once I told him … and that's ….really determined the way I feel about it …. . I imagine it's like being in the closet”. *(C9 - 40 year old British man bereaved aged 16 by suicide of brother)*

In most cases this related to suicide loss, but on probing one interviewee who described concealment of a non-suicide death, he explained that this fear related to being viewed negatively or as “weird”.

“it's not something I volunteer early on in the relationship. It might be something maybe something four to six months in.” (*D1 - 30 year old British man bereaved ten years previously by sudden unnatural death of ex-girlfriend and sudden natural death of girlfriend*)

Some used alcohol to help them broach the subject, or waited until they were very secure in their relationship, in which case disclosure was viewed as a good test of its strength.

“in a sense, it's a test for me and I've been quite lucky, but …. . I don't look forward to doing that … But, I usually have a good feeling about someone that they could probably handle it. I mean, if they don't then I'm not interested”. *(C9 - 40 year old British man bereaved aged 16 by suicide of brother)*

“I often try and mention it, you know, in the first sort of six months of knowing someone because it seems to be a bit of a bombshell to drop later on.” *(D7 - 27 year old British man bereaved aged 17 by suicide of mother)*

### 3.4 Interviewee reflexivity

Some interviewees had clear insight into the subjectivity of the stigma they experienced. For example C9 (a 40 year old British man bereaved aged 16 by suicide of his brother) observed “this is all perceived …. .the whole thing is probably slightly magnified too, because ….the anxiety is not giving me a real picture …. probably people aren't that bothered”. Regarding people's social awkwardness he commented “to be honest, I don't know how much that is in reality, or just my perception. I think my perception is probably slightly skewed, but … it's not completely in my imagination”.

## 4 Discussion

### 4.1 Main findings

Our study presents evidence to contradict the assertion that the death taboo has been overstated in Western society. Previous British work indicated that it persists in relation to violent deaths (Chapple et al., 2015). The current study provides evidence that it applies more broadly to sudden deaths, perhaps due to their shocking or unusual nature; causing others significant unease. Interviewees attributed this unease to a lack of confidence on the part of others over appropriate responses, and this clearly reinforced our interviewees' sense of social awkwardness. Our aim had been to identify whether certain dimensions of stigma were common to all three groups, or unique to particular modes of bereavement. We found evidence for both, suggesting a layering effect of different dimensions of stigma according to cause of death. Both our higher-order themes applied to all three groups. Overlying the universal experience of unease, stigma took the form of pity in relation to natural causes, and blame and shame in relation to suicide and other unnatural causes. Only the sub-themes of failure to offer support and avoidance of the word suicide were unique to suicide. For this group, accounts of extreme social awkwardness were much more common than examples of others’ negative attitudes to suicide. This was striking given the heavy emphasis on distaste and disapproval in historical reviews (Cvinar, 2005). Generally, a taboo was perceived more often in relation to displays of grief than in relation to the cause of death. However, there was also evidence of cultural variations: themes of blame and concealment of the cause were more apparent in the accounts provided by Eastern and Southern European interviewees than by British interviewees. We had expected findings to vary by gender and time since death but such differences were not apparent.

### 4.2 Results in the context of other studies

Attempts to relate these qualitative interview findings to our previously published quantitative findings of significant group differences in stigma scores in this dataset (Pitman et al., 2016b) highlight the complexity of experiences of stigma in relation to bereavement. Although our qualitative results revealed some degree of taboo in relation to all forms of sudden death, the negativity and social awkwardness encountered by those bereaved by suicide stand out as particularly acute. Interviewees bereaved by suicide experienced what they described as high levels of stigma in terms of embarrassment (their own and others), avoidance by those from whom they would have expected empathy, unwelcome degrees of pity, and a marked lack of offered support. Their responses suggested that whilst there were extensive commonalities in experiences of stigma after sudden death, they may have been experienced more acutely by those bereaved by suicide due to self-stigma.

The absence of support reported by suicide-bereaved interviewees, both in terms of perceptions of others' avoidance and failure to offer support, represents both abandonment and inequitable access to resources, and strongly reinforces self-stigma. Our quantitative work has found people bereaved by suicide to be significantly more likely to report delays in receipt of support after their loss and a lack of informal support (Pitman et al., 2017b). We cannot know whether this perceived shortfall corresponded with the actual support offered, or whether such perceptions were distorted by self-stigma. However, what remains important is the perception of being abandoned. The other dimensions of stigma described in this study also depicted a sense of isolation, even in the context of apparent social support. All interviewees described others' social embarrassment, and it was this dimension of stigma and the death taboo that exerted the strongest influence on their social behaviour. They had learnt to steer the conversation deftly away from death, sparing others from any awkwardness. As with any safety behaviour, it was self-reinforcing. Other behaviours described included withholding details of the death (to dampen morbid curiosity), hiding the true extent of grief, and concealing the cause of death outside safe relationships. Our work provides insights into the complex social interactions to be navigated after a sudden bereavement, adding to the burden of grief and loneliness. Terms such as ‘bombshell’, ‘can of worms’ or ‘stepping on eggshells’ illustrated the charged environments experienced, and the strain of the death taboo.

Our qualitative findings regarding others' avoidance complement those of a British qualitative study of GPs, in which reported hesitance in offering support to suicide-bereaved parents was explained by guilt and a lack of confidence in knowing what to say (Foggin et al., 2016). Our results are also consistent with those of one other British qualitative study comparing experiences of stigma following bereavement by suicide and by other unnatural causes, although that did not include interviewees bereaved by natural causes (Chapple et al., 2015). This study reported that interviewees bereaved by suicide, accidental death, and murder felt stigmatised and unable to mourn openly, described others' social difficulties in discussing or acknowledging the topic, and expectations to hide their grief due to social distaste over the associations of shame and blame. As with our study, it identified themes common to all those bereaved by unnatural causes, corresponding to disrupted interactions, avoidance of the topic, avoidance of the bereaved, and aversion to displays of grief. Similar to our study, interviewees perceived a societal expectation to ‘put on a brave face’ and reach rapid ‘closure’. However themes of fear, contamination, shame and blame in relation to suicide were more prominent in that dataset. Unlike our study their sample included interviewees bereaved following public disasters, such as terrorist attacks. These interviewees differed from those bereaved by suicide or accidental death, in that their public displays of grief and anger were both tolerated and expected. The authors suggested that public disasters exempted the bereaved from the social restrictions applied to those tainted by the negative associations of suicide and accidental deaths, and the implied blame. Our study lacked this perspective, presenting instead a pervasive experience of social disapprobation of grief.

Qualitative studies of suicide-bereaved people outside the United Kingdom have not included comparisons with people bereaved by other causes. Our findings are comparable to those of three Irish qualitative studies with suicide-bereaved adults, which describe experiences of social isolation (Gaffney and Hannigan, 2010), social awkwardness (Begley and Quayle, 2007), and perceived prejudice (Nic an Fhaili et al., 2016). This Irish work also made clear links between stigma and the recent decriminalisation of suicide (Gaffney and Hannigan, 2010), and between stigma and reluctance to seek help (Nic an Fhaili et al., 2016); dimensions not apparent in our dataset. Other international research on self-stigma and public stigma towards people bereaved by suicide is summarised in a recent systematic review, identifying 11 qualitative studies (Hanschmidt et al., 2016). Consistent with our findings, these studies described the stigma of suicide bereavement in relation to others’ social discomfort and avoidance, with participants describing feeling blamed or gossiped about, concealing the cause of death, and concealing their grief. Ours is therefore the only study to show that some of these themes are common to people bereaved by other causes of sudden death. Studies in this review conducted outside Britain also identified dimensions of stigma not apparent in our sample. For example, Australian work described stigma arising from religious and recent legal sanctions against suicide. Chinese work identified public perceptions of suicide as a failure of the family, stigmatising them through dishonour. Israeli, US and Australian work described stigma arising from the association between suicide and mental illness, discrediting the deceased and their family. Taiwanese, Israeli, Australian, and US studies brought out a deep sense of shame and embarrassment. This internalised (or self-) stigma was relatively absent from the experiences of our sample, who described shame or embarrassment mainly in relation to social awkwardness.

### 4.3 Strengths and limitations

We used a large national sample of bereaved adults within a defined population, followed by purposive sampling to reflect a range of experiences. Our inclusion of participants with diverse nationalities reflected the British population's cultural mix and permitted cross-cultural comparisons. We achieved reasonable gender representation given the number of men who volunteered to participate. Our in-depth interviews probed the issues of stigma and support to address specific research questions, and detailed the lived experiences of stigma following sudden bereavement. Ours is the only qualitative study to have compared experiences of people bereaved by suicide, other unnatural deaths, and deaths by natural causes (Hanschmidt et al., 2016), therefore permitting investigation of whether specific constructs were unique to one type of loss. We followed established guidelines on the design and reporting of qualitative research (Tong et al., 2007), maintaining an awareness of the influence of researcher attitudes on interviewer style and coding of responses.

Our sample was drawn from higher education settings, and respondents were predominantly white, female, and highly-educated, limiting generalisability. There was potential for response bias in that the bereaved people who had the strongest views might be those most likely to volunteer for an interview. Our dataset lacked the accounts of those who described being able to talk freely about their loss. It is possible that our topic guide elicited partial accounts, focussing on negative experiences. Our methodological approach relied on interviewees' own interpretation of social interactions, and we lacked the perspectives, attitudes and underlying motives of the social contacts they referred to. It is possible that the stigmatising social awkwardness described by those bereaved by suicide and other unnatural causes arose from an underlying aversion to violent death, rather than others' fear of saying the wrong thing. Such hidden distaste might apply after any death viewed as preventable, whether by unnatural (Chapple et al., 2015) or natural causes (Chapple et al., 2004). However, as we did not measure self-stigma, we were unable to explore whether perceptions of stigma were conditioned by individuals’ tendency to self-stigmatise in the context of societal stigma.

### 4.4 Clinical, policy and research implications

The stigma of mental illness has been conceptualised as a stressor in its own right (Rusch et al., 2014). Our findings demonstrate that each dimension of the stigma of sudden bereavement causes tangible distress and a sense of isolation. This defines the stigma of sudden bereavement as a stigma stress. Given the association between the stigma of sudden bereavement and suicide attempt (Pitman et al., 2017a), there is clearly a need to reduce this stigma or to mitigate its effects. We need to develop and trial acceptable individual-level or community-level interventions to challenge negative attitudes and taboos about talking about death in order to reduce social awkwardness, and address the barriers to seeking or receiving support. This is particularly important for people bereaved by suicide, given their elevated risk of suicide attempt (Pitman et al., 2016a). An evidence base for interventions would also promote the belief that seeking help for a problem is actually beneficial, as this has been shown to be at the core of help-seeking intentions (Schomerus and Angermeyer, 2008).

In view of the difficulties in separating out bereaved people's perceptions of stigma from the overtly stigmatising attitudes of others, it is unclear whether anti-stigma interventions would be better targeted at the bereaved or at society (Cvinar, 2005). Suggested population approaches include educating the public in appropriate ways of supporting a bereaved person. This would also serve to reduce self-stigma for those subsequently bereaved. Targeted approaches include proactive outreach from voluntary sector organisations or primary care to overcome the presumed effects of stigma on help-seeking (Rusch et al., 2014). Individual-level interventions could help bereaved individuals manage perceived and enacted stigma using adapted cognitive-behavioural approaches, such as those trialled in psychiatric settings (Knight et al., 2006). Generally an improved awareness of the needs of bereaved people and of available support sources (Public Health England & National Suicide Prevention Alliance, 2015), would reinforce the idea that support is indicated. Family doctors should be made aware that people who experience potentially traumatic bereavement may feel reluctant to disclose their loss when presenting for other reasons, and feel unworthy of help for their grief.

## 5 Conclusions

This qualitative study of the experiences of stigma described by people bereaved by suicide, other sudden unnatural deaths, and sudden natural deaths found many commonalities in accounts of stigma relating to the loss. This was primarily manifested in others' social awkwardness, which caused interviewees significant distress. In people bereaved by suicide accounts of social unease were much more common than experiences of specific negative attitudes towards them or the deceased. The only dimensions of stigma specific to people bereaved by suicide were the sub-themes of a failure to offer support and avoidance of the word suicide, both of which appeared to be driven by social awkwardness. Concealing the cause of the death and perceiving others’ blaming attitudes were common to people bereaved by suicide and other unnatural causes. Given the links between stigma and suicidality, there may be a role for individual- and community-level anti-stigma interventions after sudden bereavement, and particularly suicide bereavement. These could challenge negative attitudes, reduce social awkwardness, and address the barriers to seeking or receiving support.

## Conflicts of interest

None.

## Appendix 1 Topic guide for semi-structured interview

• Summarise interviewer's understanding of the interviewee's responses to the online questionnaire regarding their relationship to the deceased and the nature of the death
• How have people around you reacted to your bereavement?
• How easy has it been to talk about the death with the people around you?

### Impact on relationships:

• Partner/potential partners
• Close friends/potential close friends
• Immediate family
• Wider family
• Others

### Impact on other areas: (if time)

• Educational progress, Work performance, Alcohol/drugs, Finances, Spirituality or spiritual beliefs

### Other topics:

• Concealed information
• Avoidance of topic
• Hidden grief
• Fear of same death
• Memorial service
• Inquest
• Views on help offered or not offered
• Support available to others instead
• Stigmatising or honouring attitudes: What are your thoughts about society's attitude towards you because of your bereavement?

## Probes

“You've talked about a change in your (*work output/etc*), to what extent do you think this may have been due to the way your (*father/etc*) died?” “Is there anything you'd like to say with the Dictaphone off?” “If you met someone who experienced sudden bereavement how would you respond to them?”
